# The Erythrocyte Sedimentation Rate and Its Relation to Cell Shape and Rigidity of Red Blood Cells from Chorea-Acanthocytosis Patients in an Off-Label Treatment with Dasatinib

**DOI:** 10.3390/biom11050727

**Published:** 2021-05-12

**Authors:** Antonia Rabe, Alexander Kihm, Alexis Darras, Kevin Peikert, Greta Simionato, Anil Kumar Dasanna, Hannes Glaß, Jürgen Geisel, Stephan Quint, Adrian Danek, Christian Wagner, Dmitry A. Fedosov, Andreas Hermann, Lars Kaestner

**Affiliations:** 1 Theoretical Medicine and Biosciences, Saarland University, 66424 Homburg, Germany; anra98@gmx.de; 2 Experimental Physics, Saarland University, 66123 Saarbruecken, Germany; alexander.kihm@uni-saarland.de (A.K.); alexis.darras@uni-saarland.de (A.D.); greta.simionato@uni-saarland.de (G.S.); Stephan.Quint@cysmic.de (S.Q.); christian.wagner@uni-saarland.de (C.W.); 3 Translational Neurodegeneration Section “Albrecht-Kossel”, Department of Neurology, University Medical Center Rostock, University of Rostock, 18051 Rostock, Germany; kevin.peikert@med.uni-rostock.de (K.P.); Hannes.Glass@med.uni-rostock.de (H.G.); andreas.hermann@med.uni-rostock.de (A.H.); 4 Department of Neurology, Technische Universität Dresden, 01307 Dresden, Germany; 5 Institute for Clinical and Experimental Surgery, Saarland University, 66424 Homburg, Germany; 6 Institute of Biological Information Processing and Institute for Advanced Simulation, Forschungszentrum Jülich, 52425 Jülich, Germany; a.dasanna@fz-juelich.de (A.K.D.); d.fedosov@fz-juelich.de (D.A.F.); 7 Central Laboratory, Saarland University Medical Centre, 66424 Homburg, Germany; Juergen.Geisel@uks.eu; 8 Cysmic GmbH, 66123 Saarbrücken, Germany; 9 Neurologische Klinik und Poliklinik, Ludwig-Maximilians-Universität, 81366 Munich, Germany; danek@lmu.de; 10 Physics and Materials Science Research Unit, University of Luxembourg, 1511 Luxembourg, Luxembourg; 11 DZNE, German Center for Neurodegenerative Diseases, Research Site Rostock/Greifswald, 18051 Rostock, Germany; 12 Center for Transdisciplinary Neurosciences Rostock (CTNR), University Medical Center Rostock, University of Rostock, 18051 Rostock, Germany

**Keywords:** acanthocytes, dasatinib, erythrocyte sedimentation rate, 3D shapes, microfluidics, phase diagram, cell rigidity, mesoscopic modeling

## Abstract

Background: Chorea-acanthocytosis (ChAc) is a rare hereditary neurodegenerative disease with deformed red blood cells (RBCs), so-called acanthocytes, as a typical marker of the disease. Erythrocyte sedimentation rate (ESR) was recently proposed as a diagnostic biomarker. To date, there is no treatment option for affected patients, but promising therapy candidates, such as dasatinib, a Lyn-kinase inhibitor, have been identified. Methods: RBCs of two ChAc patients during and after dasatinib treatment were characterized by the ESR, clinical hematology parameters and the 3D shape classification in stasis based on an artificial neural network. Furthermore, mathematical modeling was performed to understand the contribution of cell morphology and cell rigidity to the ESR. Microfluidic measurements were used to compare the RBC rigidity between ChAc patients and healthy controls. Results: The mechano-morphological characterization of RBCs from two ChAc patients in an off-label treatment with dasatinib revealed differences in the ESR and the acanthocyte count during and after the treatment period, which could not directly be related to each other. Clinical hematology parameters were in the normal range. Mathematical modeling indicated that RBC rigidity is more important for delayed ESR than cell shape. Microfluidic experiments confirmed a higher rigidity in the normocytes of ChAc patients compared to healthy controls. Conclusions: The results increase our understanding of the role of acanthocytes and their associated properties in the ESR, but the data are too sparse to answer the question of whether the ESR is a suitable biomarker for treatment success, whereas a correlation between hematological and neuronal phenotype is still subject to verification.

## 1. Introduction

Chorea-acanthocytosis (ChAc) is a rare hereditary autosomal recessive neurodegenerative disease, caused by mutations in the *VPS13A* gene [1]. A portion of the red blood cells (RBCs) has the pathological shape of acanthocytes [2]. Symptom onset usually occurs in the third to fourth decades [3]. The interconnections between mutations, neurodegeneration and the role of the RBCs is largely elusive. In particular, it is unknown to what extent the neurological phenotype and its individual longitudinal progression may or may not be related to hematological parameters, including acanthocyte number and erythrocyte sedimentation rate [2]. However, it was found that in RBCs and iPS-derived neurons, the activity of Lyn-kinase is significantly increased [4], which was proposed to contribute to the pathophysiology of the disease [1]. Therefore, Lyn-kinase inhibitors, such as dasatinib, have moved into the focus of ChAc research. This point of view is substantiated by in vitro investigations of human RBCs [4] as well as by systemic investigations of a ChAc mouse model [5].

A first off-label approach with dasatinib indicated target engagement at least in red blood cells but—potentially due to slow disease progression—did not reveal clear treatment effects in the central nervous system [6]. Due to mild gastrointestinal side effects [7], a second treatment period with a reduced dose (from 100 to 80 mg/day) was performed.

Considering diagnosis, ChAc is often diagnosed late and is also prone to misdiagnosis due to, e.g., (i) the difficulty to identify acanthocytes in the blood smears [8] and (ii) the fact that approximately 10% of the patients do not show acanthocytes at all [9]. The prevalence of approximately 1:1,000,000 is not in favor of increasing awareness of the disease. Recently, we reported the usefulness of the ESR as a diagnostic marker. We observed that the ESR is not increased but lowered, in contrast to the common effect of inflammatory diseases. Another deviation from classical ESR parameters is the preferential reading after a sedimentation time of two hours instead of the conventional one hour. We also had the chance to analyze blood samples from the second dasatinib treatment period and compare them to samples which were taken after the end of this treatment period.

The aim of this study was to test, as previously proposed [10], whether the ESR can be used as a biomarker to monitor treatment response of dasatinib and to explain the influence of particular plasma components and cellular properties on a putative change in the ESR.

## 2. Materials and Methods

### 2.1. Blood Collection

Blood samples were collected from ChAc patients and healthy control persons. Patients were being treated at the Department of Neurology of Technische Universität Dresden. The diagnosis of ChAc was based on the clinical phenotype and was confirmed by detection of *VPS13A* mutations and by chorein Western blot [11]. Blood sample collection was approved by the “Ärztekammer des Saarlandes”, ethics votum 51/18, and performed after informed consent was obtained according to the Declaration of Helsinki. Blood was taken in the morning during routine patient visits and immediately transported to Saarland University in Homburg and Saarbrücken for further analysis, which started 6 to 8 h after withdrawal. Since transportation can have a tremendous effect on blood parameters [12,13], samples from the patients’ mother and one of the investigators were taken as transportation controls.

### 2.2. Dasatinib Treatment

Two ChAc patients (P1 and P2, Supplementary Table S1) were treated off-label with the FDA-approved tyrosine kinase inhibitor (TKI) dasatinib (DRKS00023177). Diagnosis was confirmed by chorein Western blot and genetic testing long before the study was initiated during clinical routine. The patients gave their informed consent for the off-label treatment with dasatinib. The dose of 80 mg dasatinib per day was administered orally. Evaluation of the off-label treatment primarily included monitoring of potential adverse reactions or events, as well as outcome assessments in the context of routine care adjusted to the clinical phenotype (see Reference [6]) initially every 2 weeks and after 8 weeks of treatment monthly/bimonthly.

### 2.3. Determination of the Erythrocyte Sedimentation Parameters

Erythrocyte sedimentation rate (ESR) measurements were performed in standard Westergren tubes (Dispette original, REF GS1500) of 200 mm height with Ethylene Diamine Tetraacetic Acid (EDTA) blood. Color pictures of the tubes were automatically taken every minute with a Canon EOS 500D camera for up to 50 h to cover the whole range of sedimentation in every case. For each tube, a custom-written MATLAB algorithm extracted the position of the interface at the top of the concentrated erythrocyte suspension with an accuracy of at least 0.1 mm.

### 2.4. Selection and Determination of Clinical Laboratory Parameters

Measurements of clinical laboratory parameters were performed by standard methods in the Clinical Chemistry Laboratory of Saarland University Hospital (Homburg, Germany). Since a previous study identified both RBC properties and plasma constituents as contributors to a delayed ESR [10], we tested routine RBC and plasma parameters. RBC-related parameters tested were hematocrit, RBC number, hemoglobin concentration, mean cellular volume (MCV), mean cellular hemoglobin (MCH) and mean cellular hemoglobin concentration (MCHC). The blood plasma parameters we analyzed were parameters that we could at least slightly relate to RBC aggregation, which are concentrations of total protein, albumin, fibrinogen, C-reactive protein (CRP) as well as the immunoglobulins G (IgG) and M (IgM).

### 2.5. Erythrocyte 3D Recordings and Shape Classification

Approximately 5 µL of blood was placed into 1 mL of 0.1% glutaraldehyde (Sigma Aldrich, St. Louis, MO, USA) solution in phosphate-buffered saline (PBS) in order to fix the shape of the red cells [14]. Erythrocytes (5 µL in 1 mL PBS) were stained with 5 µL of CellMask Deep Red plasma membrane stain (0.5 mg/mL; Thermo Fisher Scientific, Waltham, MA, USA) for 24 h at room temperature. Subsequently, the cells were washed 3 times by centrifugation at 4000× *g* for 5 min (Eppendorf Micro Centrifuge 5415 C, Brinkmann Instruments, Riverview, FL, USA) in 1 mL of PBS solution. After washing, the cells were resuspended in PBS and finally placed on a glass slide for confocal microscopy [15]. Each labeled sample was placed between two glass slides for imaging (VWR rectangular cover glass, 24 × 60 mm^2^) by employing a piezo stepper for a 20 µm *z*-range. Confocal image generation was performed with a spinning disk-based confocal head (CSU-W1, Yokogawa Electric Corporation, Musashino, Japan). Image sequences were acquired with a digital camera (Orca-Flash 4.0, Hamamatsu Photonics, Hamamatsu City, Japan). A custom-written MATLAB routine was used to crop single cells from each image and perform 3D reconstruction to enable visualization of the 3D shapes of the cells. Each single-cell 3D image contained 68 individual planes with an extent of 100 pixels by 100 pixels and a lateral (*x/y*) size of 0.11 µm/pixel. The piezo stepper had a minimal step width of 0.3 µm, defining the *z*-plane distance. To compensate for the difference in resolution in the *x/y* and *z*-directions, we modified the *z*-scale by means of linear interpolation. Thus, the obtained *z*-stack had dimensions of 100 × 100 × 185 voxels. The image stacks were then passed to a custom-written ImageJ script. By applying a fixed threshold for every image, the script binarized the confocal *z*-stack to retrieve the cell membrane as an isosurface. Based on these iso-surface images, an artificial neuronal network was used to classify the cell shapes, as recently described [16]. This classification was based on spherical harmonics and allowed a completely unbiased cell classification.

### 2.6. Microfluidic Measurements

The microfluidic measurements were performed by applying a set of discrete pressure drops in a regime up to 700 mbar to a connected Eppendorf tube containing the final blood solution [17]. To obtain this final blood solution, a volume of 10 µL of RBCs washed (centrifugation at 1500× *g* for 5 min) 3 times in PBS was filled to 1 mL total volume with a solution of PBS and 1 mg/mL bovine serum albumin (BSA), yielding a hematocrit of 1%. We advected this solution through microfluidic channels with a cross-section of a width of 12 µm, a height of 10 µm and a length of approximately 4 cm.

The flowing RBCs were recorded with a brightfield microscope equipped with an oil-immersion objective (Plan Apo 60 ×, Nikon, Tokyo, Japan) at a distance 10 mm away from the channel entrance to avoid the recording of transient cell shapes [18,19]. With the aid of a custom-tailored particle tracking algorithm, we extracted cropped images of single flowing RBCs together with their individual velocities and centroid positions.

Cells flowing due to a distinct pressure drop were grouped, and from this set, the mean velocity was obtained. Analogously, cells associated to one distinct pressure drop were manually categorized into the scheme of croissants, slippers and acanthocytes [20].

We analyzed the fraction of RBCs obeying healthy morphologies (i.e., slippers and croissants) and acanthocytes. Based on this classification, we fit the deformed acanthocytes fraction, *φ_Ac_*, heuristically with an exponential decay, according to *φ_Ac_(v) = N_0_* exp*(dv)*, with the velocity, *v,* and fit parameters *N_0_* and *d*. *N_0_* hereby defines the total fraction of deformed acanthocytes in stasis, and *d* is a deformability parameter.

### 2.7. Aggregate 2D Simulatioons

Two-dimensional (2D) simulations were used to study changes in characteristic hole sizes between aggregated cells for various mixtures of healthy erythrocytes, rigidified discocytes and acanthocytes, as hole sizes determine aggregate permeability. A ring-like bead-spring model with 50 vertices has been employed to represent both discocyte and acanthocyte (a flower-like shape with six rounded corners) shapes. Note that the ring circumference is the same for both shapes, while the enclosed area differs. The total potential energy of the RBC model includes three parts: (i) elastic energy of springs that sequentially connect all vertices into a ring-like configuration, (ii) bending energy between two neighboring springs to represent cell bending rigidity and (iii) an area constraint to enforce the discocyte shape for a fixed circumference [21,22]. Spring rigidity was set sufficiently large, such that membrane stretching can be neglected. The bending rigidity for healthy discocytes was equal to 50 *k*_B_*T*, and for rigidified discocytes, to 500 *k*_B_*T*. The acanthocyte shapes were assumed to be fully rigid.

Langevin dynamics with *k*_B_*T* = 1 has been employed to simulate aggregation of RBCs within a periodic domain of size 300 × 300 µm^2^. Lennard–Jones potential, *U(r)* = 4*ε*((*σ/r*)^12^ − (*σ/r*)^6^) for *r* < *r*_cut_ with *σ* = 0.3 µm and *r*_cut_ = 0.72 µm, was used to implement aggregation interactions between cells. The strength of the potential, *ε*, was varied within the range of 0–3 *k*_B_*T* in different simulations to represent various aggregation strengths. The total hematocrit was set to *φ* = 50% in all simulations.

### 2.8. Statistical Analysis

Statistical analysis was performed in Prism8 (GraphPad Software Inc., San Diego, CA, USA). All datasets were checked for normality of the distribution with the Shapiro–Wilk test. Statistical differences were evaluated with the Kruskal–Wallis test (characteristic hole size) or an unpaired Student’s t-test (transition point). The significance of *p*-values is abbreviated as ns (not significant) for *p* > 0.05, * for *p* < 0.05, ** for *p* < 0.01, *** for *p* < 0.001 and **** for *p* < 0.0001.

## 3. Results

### 3.1. The Dasatinib Off-Label Treatment

**Figure 1:**
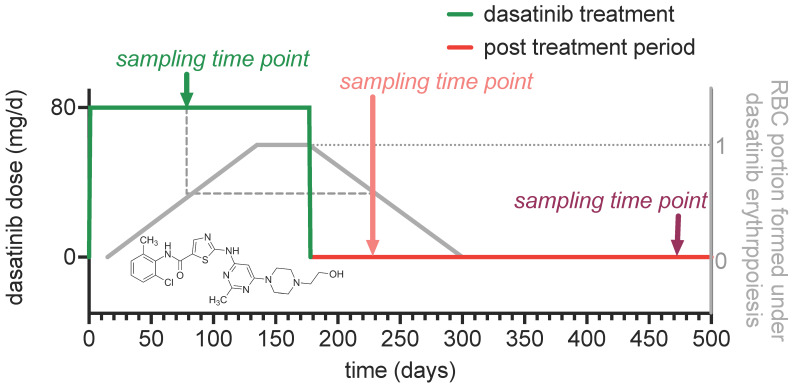
The course of the off-label treatment of two ChAc patients with dasatinib. The diagram depicts the temporal information of the treatment with the sampling time points of the measurements performed within this study. The insert shows the structural formula of the Lyn-kinase inhibitor dasatinib.

The results we report rely on a dasatinib off-label treatment of two genetically confirmed ChAc patients (P1 and P2 in Supplemental Table S1), which was performed at the end of 2019 (DRKS00023177). The evaluation and protocol of an initial treatment period with 100 mg dasatinib/day was previously published [6]. Due to mild gastrointestinal side effects [7], a second treatment period for almost six months with a reduced dose (from 100 to 80 mg/day) was performed accordingly. This was at a time when we initially started the measurements of the erythrocyte sedimentation rate (ESR) of neuroacanthocytosis syndrome (NAS) patients [10]. Therefore, we do not have pretreatment measurements but two different after-treatment time points. The timeline of the treatment and the sampling time points are given in Figure 1.

We do not know if dasatinib has a direct effect on the mature red blood cells (RBCs) or if it acts exclusively on erythropoiesis [7], which would not be unusual for drugs that manipulate kinase activity [23]. Therefore, we provide an estimation of the portion of RBCs in the circulation which were ‘produced’ in the presence of dasatinib (grey curve in Figure 1). This is based on the observation of in vivo cohort labeling experiments [24]. When glycine containing a rare isotope is orally administered, RBCs containing the labelled glycine appear in the circulation about 15 days later and show an average lifetime of 115–120 days [24]. As a result, the portion of RBCs formed under dasatinib treatment is similar for the sampling during dasatinib treatment and the first sampling after treatment (dashed line in Figure 1).

### 3.2. Determination of the Erythrocyte Sedimentation Parameters

**Figure 2:**
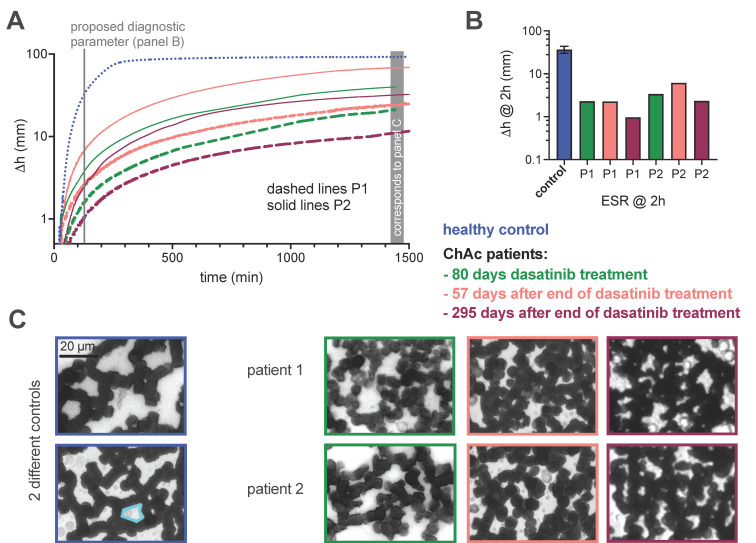
Measurements of the ESR with the standard Westergren method using EDTA blood. (**A**) Plots of the color-coded sedimentation curves retrieved from optical images taken every minute, i.e., the density of the data points corresponds approximately to the printed resolution. (**B**) Comparison of the sedimentation height after 2 h, which was proposed to be a diagnostic biomarker [10]. Please note that panels (**A**,**B**) are logarithmic plots. (**C**) Representative micrographs of sedimented RBCs forming aggregates on a coverslip. RBC are diluted in plasma, forming a hematocrit of approximately 45% in the 2D layer on the coverslip. The cyan-framed area is an example to indicate a ‘hole’.

Since the ESR was proposed as a biomarker for NAS treatments [10], we performed ESR measurements technically identical with the initial report [10] and present the results in Figure 2. This includes the sedimentation curves (Figure 2A), the sedimentation height at 2 h of sedimentation (Figure 2B) and examples of micrographs showing the RBC aggregate formation on a coverslip with a hematocrit in the 2D layer of approximately 45% (Figure 2C). We show the latter because hole size (the area of the ‘compartments’ between the RBCs) was shown to correlate with the ESR [10], and we refer to it below.

For both patients, the ESR performed under dasatinib is higher (towards control samples) than at 295 days after the end of dasatinib treatment. This increase was on average 134% for P1 and 104% for P2.

### 3.3. Evaluation of Clinical Laboratory Parameters

To complement the ESR measurements and to identify putative causes for differences in the ESR, we documented standard clinical laboratory parameters since both RBC (Supplemental Figure S1A–F) and plasma properties (Supplemental Figure S1G–L) contribute to slow sedimentation in NAS patients [10]. The diagrams in Supplemental Figure S1 show the selected parameters for all three sampling time points of the two ChAc patients investigated, with the blue dotted and dashed lines indicating the upper and lower reference values, respectively.

Since there are only single measurements, there is no option for statistical comparison. However, most parameters are within the reference value range and do not present obvious deviations along the three time points. Only P1 is slightly below the lower reference value for total protein and IgG, but these parameters do not explain the differences in ESR. Although, within the reference range, the C-reactive protein values obey temporal variations, which are not likely to explain any changes in the RBC sedimentation behavior [25].

### 3.4. Erythrocyte Shape Classification

**Figure 3:**
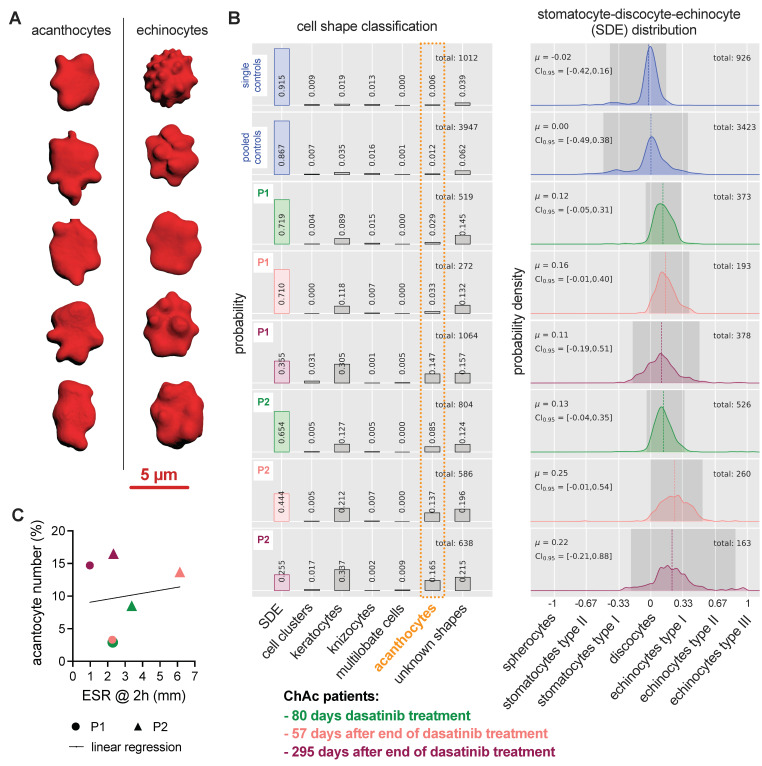
Classification of RBC morphology by an artificial neural network (ANN). (**A**) Panel A shows 3D-rendered RBCs based on confocal stack recordings. We present the comparison of acanthocytes on the left and echinocytes on the right, as classified by the ANN. (**B**) Panel B depicts the quantification of the morphological classification for a representative healthy control, a pooled control of 10 donors and the two ChAc patients for the three sampling time points. (**C**) In panel C, the acanthocyte number is plotted against the ESR for the two ChAc patients. There is no correlation between the parameters (slope of the linear regression is not significantly different from zero (*p* = 0.8)).

For shape classification of RBCs, we performed confocal imaging and 3D rendering [10], with results shown in Figure 3A, that presents acanthocytes (left column) in comparison to echinocytes (right column). The discrimination between acanthocytes and echinocytes can be challenging and is, therefore, error-prone and likely to be biased [8,10]. Thus, we used an artificial neural network (ANN) to classify cells in an unbiased, automated manner [16]. Results are shown in Figure 3B. The left part shows the first step of the classification into seven distinct classes, with acanthocytes highlighted with an orange dotted frame. SDE refers to the physiological range stretching from spherocytes and stomatocytes to discocytes and echinocytes [26], see below. Further classes are: keratocytes, knizocytes (also known as trilobes) [27], multilobate cells, unknown shapes and cell clusters. The right part of Figure 3B shows the distribution of the SDE shapes on a continuous scale from –1 to 1, indicating the correlation between the scaling number and the particular cell shape at the bottom of the panel. The dashed line presents the mean value (*µ*), and the dark grey range indicates the confidence interval (CI).

The representative control donor (data shown at the top of Figure 3B) shows predominantly discocytes (*µ* close to 0), and the pooled controls of 10 healthy donors show exactly the same properties, with the exception that the confidence interval is a bit larger. For the ChAc patients, the acanthocyte number is higher than in controls, but this also applies to other pathological shapes such as the keratocytes and cells that are classified as unknown due to their unusual morphology.

When comparing the different sampling time points of both patients, we noticed the highest acanthocyte count for the final time point 295 days after the end of the dasatinib treatment. Furthermore, it seems that the CI for the SDE distribution at the last sampling time point (without the influence of dasatinib) is broader than for the previous samples (including the representative control).

In Figure 3C, the acanthocyte number was plotted against ESR, showing no correlation (*R^2^* = 0.02). Nevertheless, it is remarkable that P1, who showed exactly the same ESR twice, displayed also almost identical acanthocyte counts.

### 3.5. Modeling of RBC Aggregation

**Figure 4:**
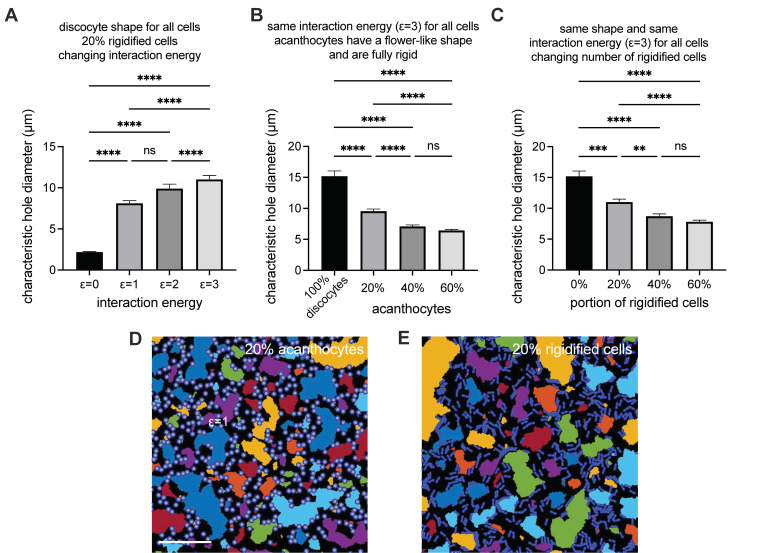
2D modeling of RBC aggregation to better understand the dependence of characteristic hole size on the aggregation strength and fraction of acanthocytes. The larger the hole size, the faster the ESR. (**A**) Panel A shows hole sizes when only the interaction energy varies. An increase in the interaction energy mimics an increase in the plasma protein concentration, mainly the fibrinogen. (**B**) Panel B represents the situation of a variable number of acanthocytes, whereas acanthocytes have a different shape and are completely rigid. (**C**) Panel C depicts the situation when the interaction energy is constant, and all cells have the same discocyte shape, but the number of rigidified cells increases. ** refers to a significance level of *p* < 0.01, *** to *p* < 0.001 and **** to *p* < 0.0001. The abbreviation ns stands for not significant. (**D**,**E**) are example images for 20% acanthocytes and 20% rigidified cells respectively, as presented in panels (**B**,**C**). For a better visualization, the holes are marked in a variety of false colors. The scale bar refers to 100 µm.

We performed mathematical modeling in order to better understand the contribution of RBC rigidity and shape to the ESR. Traditionally, inflammation and hence an increase in certain plasma proteins, mainly fibrinogen, increases RBC interaction forces, which lead to larger aggregates (conventional view [28]) or increased gel porosity (colloidal physics interpretation [10]) and therefore, result in an increased ESR. Using 2D simulations of aggregating RBCs, Figure 4A shows an increased hole size (increased ESR) with increasing interaction energy. Due to an increase in interaction forces, the cells deform and establish more compact aggregated structures, resulting in an increase of characteristic hole sizes.

Figure 4B represents similar simulations for mixtures of healthy erythrocytes and different fractions of rigid acanthocytes. Interestingly, already at 20% of abnormal cells, the characteristic hole size is significantly smaller than that of the healthy case, resulting in decreased ESR. This situation well-reflects our previously reported inverse correlation of acanthocyte number and ESR [10].

Finally, Figure 4C shows simulation results, where rigid acanthocytes are replaced by rigidified discocyte cells, and demonstrates that the shape of the acanthocytes has very little influence on hole size. Instead, it is the increased rigidity that leads to very similar values in hole size compared to Figure 4B. For illustration, panels D and E show representative images corresponding to the conditions plotted in panels B and C, respectively.

### 3.6. Erythrocyte Phase Diagram-Derived Parameters

Since the theoretical modeling revealed that for a decreased ESR, the rigidity of the RBCs is more important than the cell shape, we wanted to have a closer look at the healthy-looking RBCs (normocytes), which form croissant and slipper shapes in microfluidic flow [20]. We were especially interested in whether there was a difference in the rigidity of normocytes comparing ChAc patients (without dasatinib treatment) and healthy controls (for a detailed patient description, see Supplemental Table S1).

**Figure 5:**
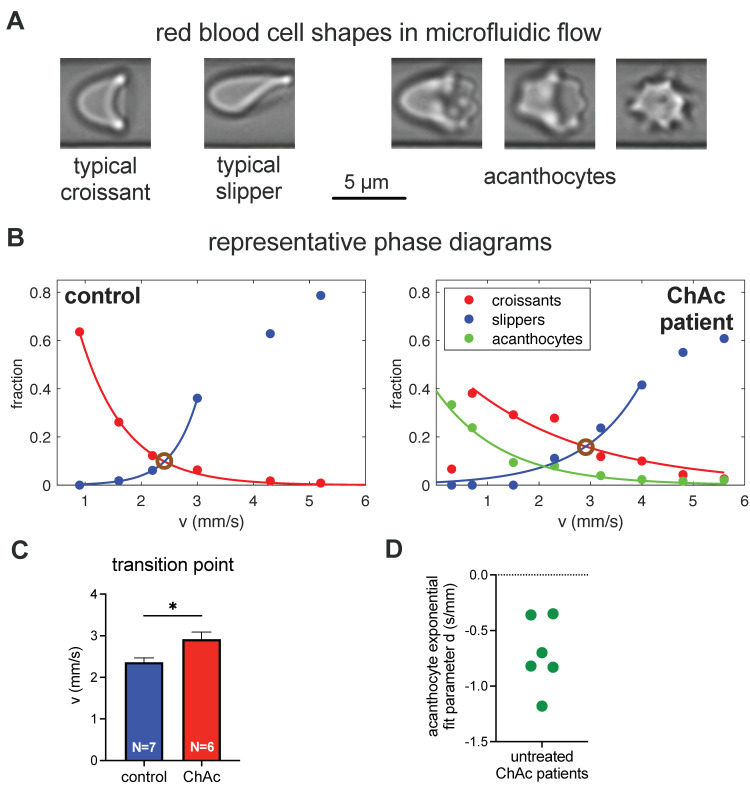
Investigation of RBC shapes in microfluidic flow. (**A**) Panel A shows the flow shapes of healthy RBCs (normocytes) on the left and a variety of acanthocytes on the right. (**B**) Panel B compares representative phase diagrams of a healthy control donor on the left and an (untreated) ChAc patient on the right, with the fitted exponential changes in the cell numbers. The transition point, where the number of croissants and slippers is identical, is marked with brown circles. (**C**) Panel C compares the transition points of a cohort of healthy controls and untreated ChAc pa-tients, indicating a higher rigidity of the normocytes in ChAc patients. * refers to a significance level of *p* < 0.05. (**D**) Panel D depicts the variance of the deformation parameter, d, of the acan-thocytes of different donors in flow.

Figure 5A presents representative images of the normocyte cell shapes of croissants and slippers (left images) in comparison to the acanthocytes in flow (right images). Figure 5B shows the phase diagram of the cell shapes (croissants, slippers and acanthocytes) as a function of the flow velocity in the microfluidic channel for a representative healthy control (left) and a representative ChAc patient (right). In the control, no acanthocytes were detected. However, in both cases, the number of croissants increases for increasing flow velocity, while the number of slippers decreases. If RBCs have an increased rigidity (decreased deformability), increased flow stresses (or larger flow velocities) are required for the transition from croissants to slippers. To obtain a quantitative measure of this transition independently of the total number of cells, we define the transition point as the speed when the number of croissants equals the number of slippers. To avoid the influence of errors in single measurement points, we performed a fit on all points within the exponential decay or the exponential growth of the croissants and slippers respectively, and determined the transition point as the intersection of the fitting curves (brown circles in Figure 5B). The statistical comparison of 6 ChAc patients and 7 healthy controls revealed a significant difference, as shown in Figure 5C. This indeed indicated a difference in the deformability, such that normocytes of ChAc patients are on average less deformable than those for healthy controls.

Furthermore, we performed an exponential fit on the fraction of acanthocytes in the phase diagram of the ChAc patients. This deformation parameter, *d,* indicates the rigidity of the acanthocytes. The plot of the *d*-value in Figure 5D shows the tremendous variability of the parameter (approximately factor of 4), indicating a significant variation in the rigidity of the acanthocytes among the ChAc patients.

## 4. Discussion

To summarize the results, the mechano-morphological characterization of RBCs from two ChAc patients in an off-label treatment with dasatinib revealed differences in the ESR and the acanthocyte count during and after the treatment period, which could not be directly related to each other. Mathematical modeling indicated that RBC rigidity is more important for delayed ESR than cell shape. Microfluidic experiments confirmed a higher rigidity in the normocytes of ChAc patients compared to healthy controls.

It is still not quite clear if dasatinib affects only RBC formation during erythropoiesis or also mature RBCs. Although there is clear evidence for processes manipulated by dasatinib during erythropoiesis [4], it is not entirely clear to what extent these putative alterations are reflected in the mature RBCs properties. With the study design used here (Figure 1), we cannot finally elucidate these effects, since our measurements are too sparse.

For the ESR, we see a compatibility between the measured values and the concept that the ESR could be a biomarker, along with other markers reflecting the disease’s manifestation in the central and peripheral nervous system, such as serum neurofilament [29], for treatment response. This is again with the reservation of an unknown relationship between hematological and neurological phenotype. Therefore, it is imperative to combine peripheral markers like the ESR with clinical and paraclinical parameters of the nervous system [6]. However, the differences detected in the ESR are not very large and the nature of an off-label treatment with two patients is not suited to provide a statistical evaluation allowing for significance testing. Further trials are required to validate the usefulness as biomarkers. Independent of that, we looked for possible reasons for the difference in the measured ESRs of the two ChAc patients, which can be caused by plasma parameters as well as by RBCs properties [10]. To this end, we performed measurements of a variety of clinical parameters (Supplemental Figure S1). Almost all values remain within the reference range and none of them were suitable to explain the differences in the ESR. Especially, the fibrinogen concentration, which is known to stabilize the RBC clusters in both stasis and flow [30,31], does not correlate with the ESR.

Although there is no correlation between the ESR and the acanthocyte count (Figure 3C), the comprehensive analysis (Figure 3B) revealed several interesting aspects. It is not just the acanthocytes that are increased in the ChAc patients (in general), but also the keratocytes and the ‘unknown shapes’. Although the network was trained carefully [16], future refinements may decrease the number of ‘unknown shapes’. Even more interesting is the RBC shape distribution among the SDE scale. Here, the ChAc patients’ samples with RBCs formed under dasatinib treatment showed a clearly narrower confidence interval than at the last sampling time point (no RBCs formed under dasatinib treatment). This is in principle in line with the measured ESR, but again it is just an initial evidence which still needs to be statistically proven. However, it is clear that RBC properties in ChAc patients need to be considered in a more holistic manner and not just in terms of acanthocyte counts.

Along this line, we performed mathematical modeling to dissect the contributions of RBCs shape and rigidity to a decreased ESR (Figure 4). The model is based on our previous work, where we could correlate the hole size in the structure of aggregating RBCs in 2D to the ESR (both in experiments and the theoretical model) [10]. Here, we showed that the rigidity of the RBCs is the dominating parameter and RBCs shape plays just a minor role. To this end, an influence of dasatinib on the cell rigidity would also be plausible. Dasatinib inhibits Lyn-kinase, which phosphorylates Band3 protein. Since Band3 protein is a structural protein, connecting the RBC membrane and the cytoskeleton [32], a reduction of the hyperphosphorylation of Band3 protein (towards ‘normal’ RBC conditions) by dasatinib would fit well into the overall picture.

Since it is known that acanthocytes have an increased rigidity [33], in our microfluidic experiments, we focused on the rigidity of the normocytes. To this end, we analyzed the transition of cell shapes from croissants to slippers in a group of seven ChAc patients (no dasatinib treatment) and seven healthy controls. A significant shift in the transition point was found (Figure 5C), confirming a higher rigidity of the normocytes in the ChAc patients. This latter outcome was not investigated in dependence of the dasatinib treatment.

## 5. Conclusions

In this paper, we characterized the mechano-morphological properties of RBCs from two ChAc patients under treatment with dasatinib. Although this provided some insights into the role of acanthocytes and their associated properties in the ESR, the data are too sparse/preliminary to answer the question of whether the ESR is a suitable biomarker to follow treatment.

## Data Availability

The data presented in this study are available in the Figures. Intermediate measurements can be obtained from corresponding authors on reasonable request.

## Supplementary Materials

The following are available online at https://www.mdpi.com/article/10.3390/biom11050727/s1, Figure S1: Clinical laboratory parameters of the two ChAc patients at different sampling time points, Table S1: Overview of the patients
