# Supplementary material for: The Erythrocyte Sedimentation Rate and Its Relation to Cell Shape and Rigidity of Red Blood Cells from Chorea-Acanthocytosis Patients in an Off-Label Treatment with Dasatinib
Source: Biomolecules. 2021 May 12;11(5):727. doi: 10.3390/biom11050727 (PMC8151862; doi:10.3390/biom11050727)
Supplement: Supplementary file 1 [file biomolecules-11-00727-s001.zip › biomolecules-1210523-supplementary.pdf]

Supplemental Material

The erythrocyte sedimentation rate and its relation to cell shape and rigidity of red blood cells from chorea-acanthocytosis patients in an off-label treatment with dasatinib

Antonia Rabe [1,2,#], Alexander Kihm [2,#], Alexis Darras [2,#], Kevin Peikert [3,4,#], Greta Simionato [2,5,#], Anil Kumar Dasanna [6,#], Hannes Glaß [3], Jürgen Geisel [7], Stephan Quint [2,8], Adrian Danek [9], Christian Wagner [2,10], Dmitry A. Fedosov [6], Andreas Hermann [3,4,11,12] and Lars Kaestner [1,2,*]

1 Theoretical Medicine and Biosciences, Saarland University, 66424 Homburg, Germany
2 Experimental Physics, Saarland University, 66123 Saarbruecken, Germany
3 Translational Neurodegeneration Section "Albrecht-Kossel", Department of Neurology, University Medical Center Rostock, University of Rostock, 18051 Rostock, Germany
4 Department of Neurology, Technische Universität Dresden, 01062 Dresden, Germany
5 Institute for Clinical and Experimental Surgery, Saarland University, 66424 Homburg, Germany
6 Institute of Biological Information Processing and Institute for Advanced Simulation, Forschungszentrum Jülich, 52425 Jülich, Germany
7 Central Laboratory, Saarland University Medical Centre, 66424 Homburg, Germany
8 Cysmic GmbH, 66123, Saarbrücken, Germany
9 Neurologische Klinik und Poliklinik, Ludwig-Maximilians-Universität, 81366 Munich, Germany
10 Physics and Materials Science Research Unit, University of Luxembourg, 1511 Luxembourg, Luxembourg
11 DZNE, German Center for Neurodegenerative Diseases, Research Site Rostock/Greifswald, 18051 Rostock, Germany
12 Center for Transdisciplinary Neurosciences Rostock (CTNR), University Medical Center Rostock, University of Rostock, 18051 Rostock, Germany
* Correspondence: lars_kaestner@me.com
# These authors contributed equally to this work.

Supplemental Table 1. Overview of the patients. P1 and P2 (green background) were temporarily treated with dasatinib. (Pro Re Nata (PRN): administration timing of prescribed medication is left to the patient/caregiver.)

**subjects**	**sex**	**age** (years)	**main clinical characteristics**	**disease duration** (years)	**medication**
P1	male	34	drug resistant epilepsy, mild chorea, tics, cognitive impairment, peripheral neuropathy, myopathy	11	lacosamide 550 mg/day, zonisamide 300 mg/day, perampanel 4 mg/day, vitamin D, PRN: lorazepam/midazolam [Dasatinib 80 mg/day]
P2	male	29	drug resistant epilepsy, mild chorea, tics, cognitive impairment, irritability, anxiety, depression, psychosis	15	lacosamide 600 mg/day, zonisamide 400 mg/day, mirtazapine 15 mg/day, olanzapine 2.5 mg/day, vitamin D, PRN: lorazepam/midazolam [Dasatinib 80 mg/day]
P3	male	53	parkinsonism, dystonia, dysarthria, peripheral, neuropathy, depression	15	scopoderm transdermal therapeutic system/day
P4	female	51	epilepsy, parkinsonism, dystonia, dysarthria, peripheral neuropathy, cognitive impairment	30	levetiracetam 4 g/day, valproate 2 g/day, clobazam 10 mg/day, zonisamide 200 mg/day, vitamin D
P5	male	54	epilepsy, parkinsonism, dystonia, dysarthria, dysphagia, peripheral neuropathy, cognitive impairment	21	lamotrigine 110 mg/day, oxcarbazepine 1.5 g/day, lacosamide 300 mg/day, levodopa 300 mg/day, esomeprazole 40 mg/day
P6	male	42	epilepsy, feeding dystonia, orofacial dyskinesia, chorea, peripheral neuropathy, myopathy, impulse control disorder	12	levetiracetame 1 g/day, quetiapine 400 mg/day, ramipril 2.5 mg/day, metoprolol 47.5 mg/day, PRN: metamizole, ibuprofen, pantoprazole

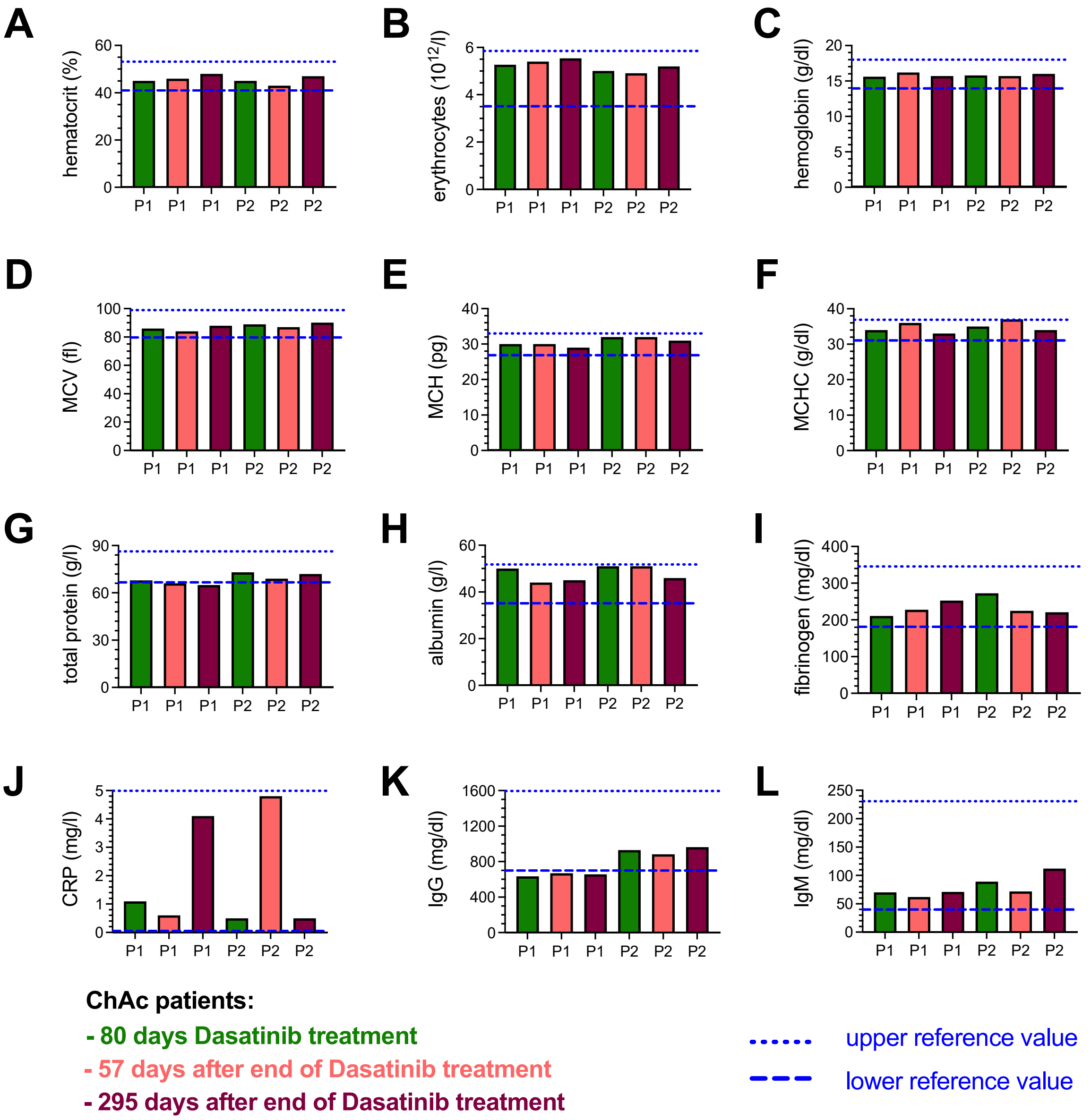

Supplemental Figure S1. Clinical laboratory parameters of the two ChAc patients at different sampling time points as indicated by the panel legend. The blue lines represent healthy reference values. The dotted lines mark the upper and the dashed line the lower limits of normal, respectively. (**A**) Hematocrit value, (**B**) Erythrocyte number, (**C**) Hemoglobin concentration, (**D**) Mean cellular volume (MCV), (**E**) Mean cellular hemoglobin (MCH), (**F**) Mean cellular hemoglobin concentration (MCHC), (**G**) Total plasma protein, (**H**) Plasma albumin, (**I**) Plasma fibrinogen, (**J**) C-reactive protein (CRP), (**K**) Immunoglobulin G (IgG), (**L**) Immunoglobulin M (IgM).
